# Supplementary material for: Exploring the Therapeutic Potential of Phosphorylated Cis-Tau Antibody in a Pig Model of Traumatic Brain Injury
Source: Biomedicines. 2023 Jun 24;11(7):1807. doi: 10.3390/biomedicines11071807 (PMC10376756; doi:10.3390/biomedicines11071807)
Supplement: Supplementary file 1 [file biomedicines-11-01807-s001.zip › biomedicines-2403118-supplementary.pdf]

Supplementary

Exploring the Therapeutic Potential of Phosphorylated *Cis*-Tau Antibody in a Pig Model of Traumatic Brain Injury

Table S1. Difference in change in ln(biomarker) over 14 days post-injury between treatment groups. FA = fractional anisotropy, MD = mean diffusivity.

	**Difference in Change between Vehicle and PNT001 Groups (95% CI), *p*-value**	
CSF Biomarkers		
NfL	−0.002 (−0.006, 0.001), $p = 0.185$	
GFAP	0.001 (−0.003, 0.007), $p = 0.487$	
UCHL-1	−0.001 (−0.003, 0.002), $p = 0.724$	
Tau	−0.001 (−0.003, 0.001), $p = 0.292$	
Plasma Biomarkers		
NfL	−0.111 (−0.554, 0.333), $p = 0.625$	
GFAP	−0.883 (−1.504, −0.261), $p = 0.005$	
UCHL-1	0.001 (0.000, 0.036), $p = 0.729$	
MRI DTI Biomarkers	Difference in Change Between Vehicle and Sham Groups (95% CI), *p*-value	Difference in Change Between PNT001 and Sham Groups (95% CI), *p*-value
FA Corpus Callosum	0.0002 (−0.0006, 0.0009), $p = 0.704$	0.0002 (−0.0006, 0.0010), $p = 0.601$
FA Left Cerebral Peduncle	0.0003 (−0.0004, 0.0007), $p = 0.373$	0.0005 (−0.0003, 0.0012), $p = 232$
FA Right Cerebral Penduncle	0.0006 (−0.0003, 0.0015), $p = 0.204$	−0.0002 (−0.0003, 0.0015), $p = 0.738$
FA Left Corona Radiata	0.0006 (−0.0002, 0.0012), $p = 0.131$	0.0007 (0.0000, 0.0014), $p = 0.055$
FA Right Corona Radiata	−0.0001 (−0.0010, 0.0009), $p = 0.886$	0.0002 (−0.0008, 0.0012), $p = 0.695$
MD Corpus Callosum	−0.0004 (−0.0012, 0.0005), $p = 0.429$	0.0003 (−0.0006, 0.0012), $p = 0.466$
MD Left Cerebral Peduncle	0.0004 (−0.0003, 0.0011), $p = 0.258$	0.0004 (−0.0004, 0.0011), $p = 0.331$
MD Right Cerebral Peduncle	0.0005 (−0.0001, 0.0011), $p = 0.098$	0.0005 (−0.0001, 0.0011), $p = 0.104$
MD Left Corona Radiata	0.0003 (−0.0002, 0.0007), $p = 0.246$	0.0004 (−0.0001, 0.0008), $p = 0.094$
MD Right Corona Radiata	0.0002 (−0.0004, 0.0008), $p = 0.511$	0.0002 (−0.0004, 0.0007), $p = 0.919$

Table S2. Plasma and CSF biomarker correlation at acute time point (first row) and subacute time point (second row). Given the different time points in which samples were collected for plasma and CSF, we chose the closes matching times with plasma (30 min) and CSF (1 h) for the acute time point. The second row shows the correlation values at 14 days.

	**NfL**	**GFAP**	**UCHL-1**
**30 min – 1 h**	$\Gamma = 0.2026$ ($p = 0.3785$)	$\Gamma = 0.2078$ ($p = 0.3661$)	$\Gamma = 0.5165$ ($p = 0.0586$)
**14 days**	$\Gamma = 0.4175$ ($p = 0.0670$)	$\Gamma = -0.1221$ ($p = 0.5980$)	$\Gamma = 0.1923$ ($p = 0.5291$)

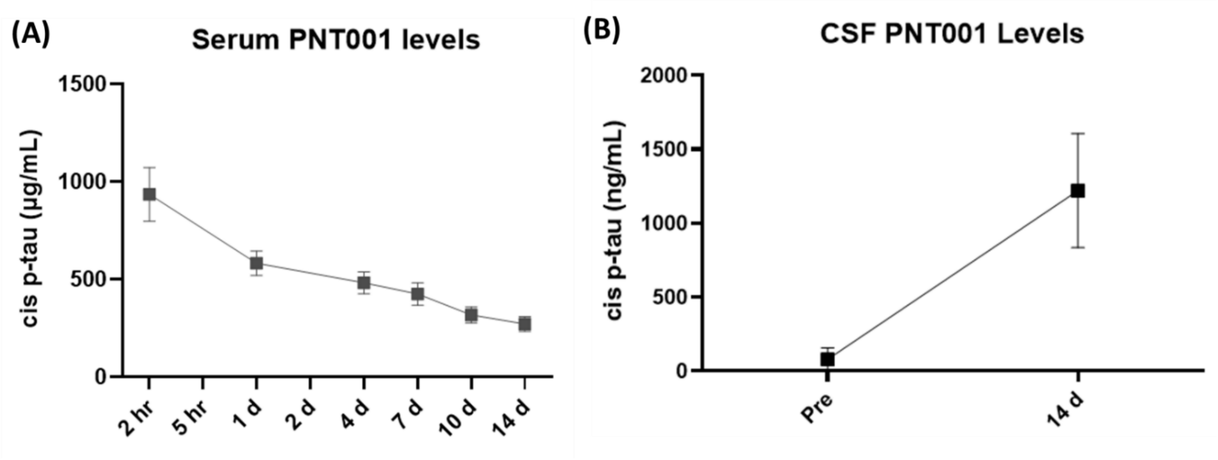

Figure S1. Exposure Data for serum (**A**) and CSF (**B**). Serum PNT001 levels are shown here, with expected decrease over time from 2 h to 14 days. Two time-point CSF levels of PNT001 was measured: prior to the injury, and at 14 days. PNT = PNT001.
